# Thyroid lesions incidentally detected by ^18^F-FDG PET-CT — a two centre retrospective study

**DOI:** 10.2478/raon-2014-0039

**Published:** 2015-03-25

**Authors:** Jan Jamsek, Ivana Zagar, Simona Gaberscek, Marko Grmek

**Affiliations:** 1 Faculty of Medicine, University of Ljubljana, Ljubljana, Slovenia; 2 Department for Nuclear Medicine, Institute of Oncology Ljubljana, Ljubljana, Slovenia; 3 Department for Nuclear Medicine, University Medical Centre Ljubljana, Ljubljana, Slovenia

**Keywords:** thyroid, ^18^F-FDG, PET-CT, PET incidentaloma, thyroid cancer

## Abstract

**Background.:**

Incidental ^18^F-FDG uptake in the thyroid on PET-CT examinations represents a diagnostic challenge. The maximal standardized uptake value (SUV_max_) is one possible parameter that can help in distinguishing between benign and malignant thyroid PET lesions.

**Patients and methods.:**

We retrospectively evaluated ^18^F-FDG PET-CT examinations of 5,911 patients performed at two different medical centres from 2010 to 2011. If pathologically increased activity was accidentally detected in the thyroid, the SUV_max_ of the thyroid lesion was calculated. Patients with incidental ^18^F-FDG uptake in the thyroid were instructed to visit a thyroidologist, who performed further investigation including fine needle aspiration cytology (FNAC) if needed. Lesions deemed suspicious after FNAC were referred for surgery.

**Results.:**

Incidental ^18^F-FDG uptake in the thyroid was found in 3.89% — in 230 out of 5,911 patients investigated on PET-CT. Malignant thyroid lesions (represented with focal thyroid uptake) were detected in 10 of 66 patients (in 15.2%). In the first medical centre the SUV_max_ of 36 benign lesions was 5.6 ± 2.8 compared to 15.8 ± 9.2 of 5 malignant lesions (*p* < 0.001). In the second centre the SUV_max_ of 20 benign lesions was 3.7 ± 2.2 compared to 5.1 ± 2.3 of 5 malignant lesions (*p* = 0.217). All 29 further investigated diffuse thyroid lesions were benign.

**Conclusions.:**

Incidental ^18^F-FDG uptake in the thyroid was found in 3.89% of patients who had a PET-CT examination. Only focal thyroid uptake represented a malignant lesion in our study — in 15.2% of all focal thyroid lesions. SUV_max_ should only serve as one of several parameters that alert the clinician on the possibility of thyroid malignancy.

## Introduction

**FIGURE 1.**
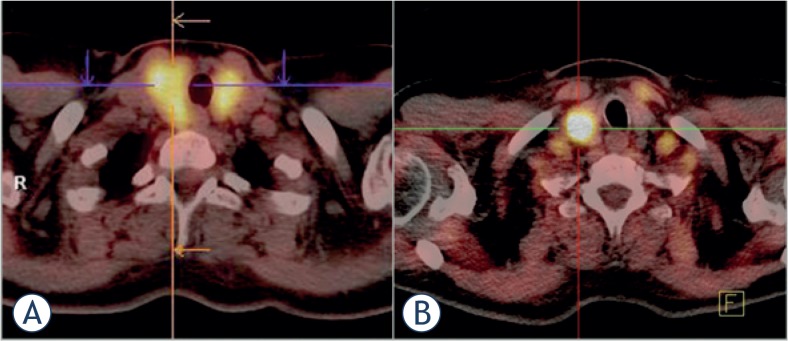
Fusion (PET-CT) scans of the thyroid. A diffuse ^18^F-FDG accumulation in the thyroid is presented on scan **(A)** (this scan was done at the Institute of Oncology Ljubljana). A focal ^18^F-FDG accumulation in the thyroid is presented on scan **(B)** (this scan was done at the University Medical Centre Ljubljana).

Incidental uptake of ^18^F-fluorodeoxyglucose (^18^F-FDG) in the thyroid is sometimes found during positron emission tomography - computed tomography (PET-CT)^1^–^3^, which is mostly used in cancer staging and diagnostics.^4^–^6^ Throughout the literature the reported incidence of incidental thyroid uptake of ^18^F-FDG on PET-CT varies between 0.2% and 8.9%.^2^ Thyroid lesions on PET-CT can be either diffuse or focal (Figure 1). Diffuse ^18^F-FDG uptake is usually associated with autoimmune thyroiditis or Graves’ disease^7^–^9^, whereas focal ^18^F-FDG uptake can be either due to a benign or malignant process in the thyroid.^10^–^19^

A semi-quantitative parameter that could help in differentiating thyroid lesions on PET-CT is the standardized uptake value (SUV), often expressed as the maximal SUV (SUV_max_) or mean SUV (SUV_mean_).^20^ However, the discriminating power of this parameter is still unclear, as some studies have reported a statistically significant difference between SUV values of benign and malignant thyroid lesions^13^,^16^,^21^,^22^, whilst others have shown no statistically significant difference.^17^,^23^–^28^ Moreover, the SUV of benign and malignant thyroid lesions varied greatly between these studies. We also know that the calculated SUV is highly dependent on the scanner type, reconstruction algorithms and software packages used, which prevents the comparisons of studies conducted at different centres using different equipment.^29^–^31^ This represented a challenge for our study.

The aims of this study were to (i) determine the incidence of thyroid lesions incidentally found on ^18^F-FDG PET-CT, (ii) identify what diffuse and focal thyroid lesions represent, and (iii) what is the optimal SUV_max_ that can discriminate between benign and malignant focal thyroid lesions incidentally found on PET-CT. This study was conducted at two PET-CT centres (having different PET-CT scanners) in Slovenia: the Department of nuclear medicine at the University Medical Centre Ljubljana (UMC) and the Institute of Oncology Ljubljana (IO).

## Patients and methods

### Subjects and study design

We retrospectively evaluated the medical records of 5,911 patients (2,840 patients from UMC and 3,071 patients from IO) who underwent an ^18^F-FDG PET-CT investigation between January 2010 and December 2011. Only patients (males and non-pregnant females) aged 18 years or more were included in this study. The ^18^F-FDG PET-CT investigation of patients included in the study was performed for different purposes, mainly because of oncologic indications. The study was approved by the Ethics Committee at the Ministry of Health, Republic of Slovenia (No.: 53/04/12).

### Methods employed

Patients from both centres fasted for at least 6 hours, ideally having a blood glucose level less than 7 mmol/l, before receiving 370 MBq of ^18^F-FDG. The acquisition on the PET-CT scanner started 60 minutes after the radiotracer administration. The PET-CT scanners used were different: at UMC a Siemens Biograph mCT and at IO a Philips Gemini 16 GXL. In all patients, the localisation and attenuation correction CT was first done, followed by the PET scan itself. The CT acquisition parameters in both centres were fairly similar. Also, the PET acquisition parameters did not differ a lot; at UMC a bed position of 2 min with 45% overlap and at IO a bed position of 2 min with 50% overlap was used. The acquired PET-CT data was processed using similar iterative reconstruction algorithms.

Nuclear medicine doctors at both centres used visual and semi-quantitative data analysis (SUV_max_) for creating a final report. They had access to relevant patient history and previous examination reports. Patients with thyroid lesion incidentally found on ^18^F-FDG PET-CT were referred to a thyroidologist.

Thyroid investigation normally included the patient’s history, clinical examination, relevant laboratory workup, ultrasound examination and ^99m^Tc scintigraphy of the thyroid. For a final diagnosis of suspicious thyroid lesions, patients were further investigated using fine needle aspiration cytology (FNAC). A histological report was obtained for lesions that were surgically removed. All data (PET-CT reports, reports of thyroid examinations, cytological and histological reports) were obtained only from patients treated and followed-up at UMC and IO.

### Statistical analyses

Statistical analysis was performed using IBM SPSS Statistics 22.0 and Microsoft Excel for Mac 14.1. The SUV_max_ of benign and malignant thyroid lesions were compared using Student’s *t*-test. Results were deemed statistically significant for *p* < 0.05. A receiver operating characteristic (ROC) analysis was performed to determine a SUV_max_ cut-off point that differentiates between suspicious and unsuspicious focal thyroid lesions.

## Results

### Characteristics of patients

The mean age of 2,840 patients who had a PET-CT investigation at UMC was 61.2 ± 12.9 years; the mean age of 3,071 patients at IO was 64.4 ± 12.1 years. Fifty per cent of UMC patients were males and 50% females. The percentage of males and females in the IO group was 52.5% and 47.5% respectively. Patients at UMC underwent an ^18^F-FDG PET-CT investigation mainly for cancer-related diagnostics or inflammatory/infection problems. On the other side, patients at IO underwent an ^18^F-FDG PET-CT investigation almost exclusively because of cancer-related diagnostics.

### Incidentally detected thyroid lesions

Incidental ^18^F-FDG uptake in the thyroid was found in 230 out of 5,911 investigated patients (in 3.89%). Focal thyroid uptake represented 64.3% and diffuse thyroid uptake 35.7% of detected thyroid lesions. 56.1% of all focal lesions and 81.7% of all diffuse lesions were detected in female patients. More detailed information about patients with incidentally found thyroid lesions on ^18^F-FDG PET-CT is presented in Table 1.

Data of further treatment were found for 58 out of 82 patients (in 70.7%) with increased ^18^F-FDG uptake in the thyroid investigated at UMC and for 46 out of 148 patients (in 31.1%) investigated at IO. Diffuse thyroid lesions in 14/58 patients (24.1%) from UMC (SUV_max_ range from 3.5 to 10.3) and in 15/46 (32.6%) patients from IO (SUV_max_ range from 1.9 to 9.2) were all benign. Hashimoto’s thyroiditis was diagnosed in 92.9% and 73.3% respectively.

At UMC, 44 patients with focal ^18^F-FDG uptake in the thyroid (SUV_max_ range from 2.3 to 31.9) were further investigated. Thyroid nodules were found in 30 patients (in 68.2%). Autoimmune thyroid disease was diagnosed in 29.5% – in 12 patients with Hashimoto’s thyroiditis and in one patient with Graves’ disease. One patient was diagnosed to have benign diffuse goitre. FNAC was performed in 28 of 44 patients (63.6%). Results of FNAC are presented in Table 2.

Out of 31 focal thyroid lesions diagnosed on PET-CT in patients from IO (SUV_max_ range from 1.5 to 8.7) thyroid nodules were found in 28 cases (in 90.3%). In two patients the focal lesion was caused by Hashimoto’s thyroiditis and in one by Graves’ disease. FNAC diagnostics were performed in 24 of 31 patients (77.4%) (Table 2).

The optimal SUV_max_ cut-off point for differentiating between suspicious and unsuspicious focal thyroid lesions incidentally detected on PET-CT, calculated using ROC analysis, was 5.4 for patients investigated at UMC (sensitivity 76.9%, specificity 61.3%, AUC = 0.785); the optimal differentiating SUV_max_ for patients investigated at IO was 4.0 (sensitivity 66.7%, specificity 73.7%, AUC = 0.754).

### Surgically removed focal thyroid lesions

Malignant thyroid disease was found in 10 out of 18 patients (55.6%) who underwent surgery. Malignant thyroid disease was more common in males (8 cases) than in females (2 cases). Nine patients with focal thyroid lesions who were referred for surgery were lost to follow-up. Therefore in 10 out of 66 patients (15.2%) with focal thyroid lesion incidentally detected on ^18^F-FDG PET-CT malignant thyroid disease was confirmed. Detailed characteristics of all surgically removed thyroid lesions are presented in Table 3.

### SUV_max_ of malignant and benign focal thyroid lesions

**FIGURE 2.**
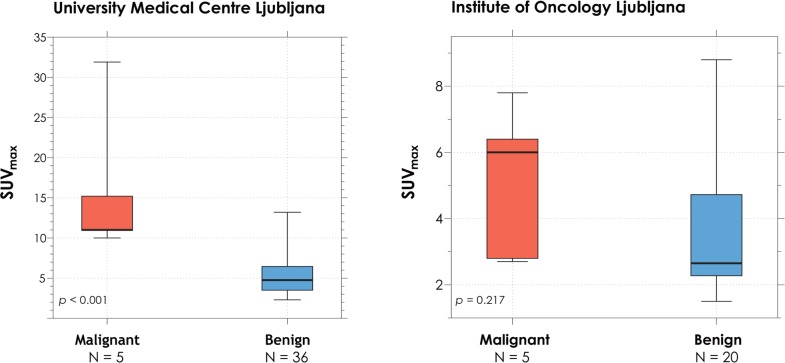
SUV_max_ of malignant and benign focal thyroid lesions (median, IQR and MIN/MAX values).

SUV_max_ of malignant focal lesions (histologically confirmed) was compared to SUV_max_ of benign focal lesions (the benign nature of a lesion was established either after a thorough thyroid examination with ultrasound, FNAC or surgical treatment) (Figure 2). A statistically significant (*p* < 0.001) difference was observed between 36 benign (SUV_max_ from 2.3 to 13.2) and 5 malignant (SUV_max_ from 10 to 31.9) focal thyroid lesions incidentally detected on PET-CT in patients from UMC. No statistically significant difference (*p* = 0.217) was observed between 20 benign (SUV_max_ from 1.5 to 8.8) and 5 malignant (SUV_max_ from 2.7 to 7.8) focal thyroid lesions in patients from IO.

## Discussion

Incidental ^18^F-FDG uptake in the thyroid was observed in 3.89% of 5,911 patients investigated; in 2.89% of patients investigated at UMC and in 4.82% of patients investigated at IO. This is in accordance with the present literature, where the incidence of such lesions varied from 0.2 to 8.9%, with most studies reporting a incidence between 2 and 3%.^2^,^3^,^11^–^13^,^16^,^17^,^19^,^21^–^28^,^32^–^36^ In a review article by Bertagna *et al.*^2^, the authors postulated that this variability in incidence could be attributed to population characteristics and background risk of thyroid disease related to specific geographic areas.

Slovenia, although not an endemic goitre region, still has a significant incidence of thyroid nodules in the general population.^37^ This could in part explain the slightly higher incidence of thyroid lesions incidentally found on PET-CT compared to some studies, where authors found a smaller incidence of thyroid lesions.^11^,^12^,^17^

According to the *American Thyroid Association Guidelines Taskforce*^38^ further investigation of incidentally found thyroid nodules is recommended. Adhering to these guidelines, all patients from our practices with an incidentally detected thyroid lesion on PET-CT were referred to a thyroidologist. Due to different reasons, not all patients had a consultation, mainly because of the management of their primary illness. In our study, 71% of patients from UMC and only 31% of patients from IO received additional thyroid diagnostics. Our explanation for this difference is that PET-CT examinations in patients at IO were done almost exclusively for staging of known primary malignant diseases – many of these patients had more severe primary malignancies that required more prompt treatment than potential thyroid neoplasms. In comparison at UMC, approximately one third of PET-CT examinations were done for non-oncologic indications in which cases additional thyroid diagnostics were more likely than in oncologic patients with more severe primary disease. Other studies also reported a similar percentage of patients with incidentally discovered thyroid PET lesions who were further investigated, with follow-up rates in the ranks of 50%.^11^–^13^,^16^–^18^,^23^–^25^,^28^

Experts agree that diffuse thyroid uptake of ^18^F-FDG on PET-CT is associated with Hashimoto’s thyroiditis.^9^ This was also confirmed by our results, where most diffuse lesions were caused by Hashimoto’s thyroiditis and no malignancy was found in patients with diffuse thyroid PET lesions.

According to the literature, the rate of focal lesions ranges from 14% to 73% of all thyroid PET lesions^8^,^16^,^24^,^32^ with a risk of malignancy in further investigated lesions of about 33%.^2^,^38^ In our study, focal thyroid lesions were present in 64.3% of all cases with incidental thyroid uptake. These lesions represented a thyroid nodule in 68.2% (UMC patients) and in 90.3% (IO patients). We histologically confirmed thyroid malignancy in 5 of 10 surgically treated patients from UMC and in 5 of 8 patients from IO. Altogether, malignant disease was observed in 10 of 66 patients (in 15.2%) with a focal ^18^F-FDG uptake in the thyroid. In comparison to other reports, the incidence of thyroid malignancy in our study was somewhat lower.^2^,^12^,^13^,^16^,^17^,^21^–^28^,^34^ This is, in our opinion, mainly due to higher goitre prevalence in our population.^37^

Autoimmune thyroid disease was present in 29.5% of focal thyroid lesions from UMC patients. This finding is quite different from data published in the literature.^18^,^23^ Our explanation for this discrepancy is in the different diagnostic process that was used in different institutions. At UMC, a thorough thyroid examination with relevant laboratory workup and an ultrasound examination of the thyroid, irrespective of the use of FNAC, was in most patients enough to make a final diagnosis of thyroid disease. The decision regarding FNAC examination was undertaken by the consulting thyroidologist on a patient by patient basis. Most studies, like the one conducted by Chu *et al.*^12^, were more in line with the IO group, where only 3 of 31 focal PET lesions proved to be of autoimmune origin.

According to the literature, Graves’ disease is demonstrated most commonly by diffusely increased ^18^F-FDG uptake in the thyroid.^39^,^40^ However, in our study, we found two cases of Graves’ disease with focal ^18^F-FDG uptake.

One of the main goals of our study was to determine whether it would be possible to differentiate between benign and malignant thyroid lesions using SUV_max_. The literature is quite divided on this topic, with studies claiming to being able to differentiate between benign and malignant lesions^13^,^21^,^22^,^41^ and others whose conclusions were the exact opposite.^17^,^23^–^28^ This was also the case in our study, where the UMC group presented a statistically significant difference between benign and malignant lesions, whereas no such difference was found in the IO group. Even though the mean SUV_max_ of malignant lesions were on average higher than benign lesions, the overlap between both sets of lesions was considerable. For example, a Hürthle adenoma had a relatively high SUV_max_ of 8.9 while on the other side; a papillary thyroid carcinoma had a SUV_max_ of only 2.8.

It should also be noted, that calculated SUV_max_ is highly dependent on the type of PET-CT scanner, reconstruction algorithms and software packages used^20^,^29^–^31^, as was the case in our study, which included two centres with different equipment. The newer Siemens Biograph^®^ mCT used at UMC had a better detector system and time of flight technology compared to the older Philips Gemini 16 GXL. These might be some of the factors resulting in different SUV_max_ readings at both centres. Therefore, the SUV_max_ of a thyroid lesion should only serve as one of several parameters that alert the clinician on the possibility of thyroid malignancy. The correct protocol in this situation is, as recommended by the *American Thyroid Association Guidelines*, to promptly investigate all focal thyroid PET lesions with additional diagnostics.^38^

## Conclusions

Incidental ^18^F-FDG uptake in the thyroid on PET-CT was found in 3.89%. Only focal thyroid uptake represented a malignant lesion in our study – in 15.2% of all focal thyroid lesions. SUV_max_ should only serve as one of several parameters that alert the clinician on the possibility of thyroid malignancy and as such must be used with caution in the interpretation of PET-CT studies.

## Figures and Tables

**TABLE 1.** Patients and characteristics of incidental ^18^F-FDG uptake in the thyroid detected by PET-CT

	**Patients with incidental thyroid uptake**			**Incidental thyroid uptake**	

	**Number (m/f)**	**Incidence (%)**	**Age (year) (average ± SD)**	**Type**	**SUV_max_ (average ± SD)**
**UMC**	61 (24/37)	2.15	63.6 ± 12.1	Focal	6.6 ± 4.4
	21 (4/17)	0.74	57.5 ± 14.4	Diffuse	7.9 ± 4.0
**(all)**	**82 (28/54)**	**2.89**	**62 ± 12.9**		**6.9 ± 4.3**
**IO**	87 (41/46)	2.83	64.2 ± 12.3	Focal	4.2 ± 2.1
	61 (11/50)	1.99	64.9 ± 11.2	Diffuse	4.3 ± 2.7
**(all)**	**148 (52/96)**	**4.82**	**64.5 ± 11.8**		**4.2 ± 2.3**

UMC = University Medical Centre Ljubljana; IO = Institute of Oncology Ljubljana; SUV_max_ = maximal standardised uptake value

**TABLE 2.** Results of fine needle aspiration cytology for focal thyroid lesions, classified according to the Bethesda classification

**Centre**	**FNAC (No.)**	**ND or UnS (No. (%))**	**BEN (No. (%))**	**AUS or FLUS (No. (%))**	**FN (No. (%))**	**SM (No. (%))**	**M (No. (%))**
**UMC**	28	2 (7.1)	17 (60.8)	2 (7.1)	5 (17.9)	0	2 (7.1)
**IO**	24	5 (20.8)	7 (29.2)	1 (4.2)	3 (12.5)	3 (12.5)	5 (20.8)
**All**	52	7 (13.4)	24 (46.2)	3 (5.8)	8 (15.4)	3 (5.8)	7 (13.4)

FNAC = fine needle aspiration cytology, ND or UnS = non-diagnostic or unsatisfactory; BEN = benign; AUS or FLUS = atypia of undetermined significance or follicular lesion of undetermined significance; FN = follicular neoplasms and oncocytic tumours; SM = suspicious for malignancy; M = malignant

**TABLE 3.** Characteristics of surgically removed thyroid lesions

**Centre**	**Referral diagnosis**	**Sex (m/f)**	**Age (year)**	**SUV_max_**	**Size (mm)**	**Cytology**	**Histology**
**UMC**	Gastric carcinoma	f	71	5.5	10	Oncocytic cells	Hürthle adenoma
	Suspicious lesion in the right lungs	m	68	4.8	12	Unsatisfactory	Nodular goitre
	Tumour of the cardia	f	48	8.9	9	Oncocytic cells	Hürthle adenoma
	Erythema nodosum and pharyngitis	f	40	7.5	22	Unsatisfactory	Hürthle adenoma
	Pelvic inflammatory disease	f	61	6.4	10	Oncocytic cells	Nodular goitre
	Lung carcinoma	m	70	15.2	30	Oncocytic cells	Follicular carcinoma
	Origo ignota malignant disease	m	48	11	21	Atypia of undetermined significance	Medullary carcinoma
	Histiocytosis	m	41	11	10	Papillary carcinoma	Papillary carcinoma
	GIT malignancy	f	64	31.9	52	Atypia of undetermined significance	Papillary carcinoma
	Metastatic lesion on the left side of the neck	m	74	10	30	Planocelluar metastasis	Planocellular subglottic carcinoma — metastasis
**IO**	Hodgkin’s lymphoma	f	64	3.2	15	Suspicious for malignancy (follicular or Hürthle)	Hyperplastic follicular benign nodule
	Malignant melanoma	m	71	2	23	Suspicious for malignancy (follicular or papillary)	Multinodular colloid goitre
	Tumour of the GE junction	f	62	8.7	35	Oncocytic cells	Hürthle adenoma
	Tumour mass in the thigh	m	22	7.8	9	Papillary carcinoma	Follicular carcinoma
	Rectal carcinoma	m	71	2.7	40	Suspicious for follicular malignancy	Follicular carcinoma
	Malignant melanoma	f	55	6	10	Papillary carcinoma	Thyroid malignancy with elements of follicular, papillary and Hürthle carcinoma
	Rectal carcinoma	m	59	6.4	10	Papillary carcinoma	Papillary carcinoma
	Rectal carcinoma	m	58	2.8	15	Oncocytic cells	Papillary carcinoma

UMC = University Medical Centre Ljubljana; IO = Institute of Oncology Ljubljana; SUV_max_ = maximal standardised uptake value; GE = gastro-oesophageal; GIT = gastro-intestinal tract
